# WHAT NEEDS TO BE DONE TO PREVENT ELDER ABUSE IN JAPAN?

**DOI:** 10.1093/geroni/igac059.1357

**Published:** 2022-12-20

**Authors:** TsuAnn Kuo, Georgia Anetzberger

## Abstract

This symposium first introduces the Japanese Act on the Prevention of Elder Abuse, including its elder abuse definitions and trends in elder abuse in both institutional and domestic settings. It then describes a recent amendment to operational standards made by ministerial ordinance, which aims to reduce elder abuse. Second, it presents analysis of elder abuse data (N=497) from Matsudo-City in Chiba prefecture, which found that only about 65% of reported elder abuse cases were deemed to meet the legal definition of elder abuse and the rest were excluded due to how the law defines abusers. Findings suggest the definitions in the Act be broadened to encompass a wider set of abuse situations. Third, it presents analysis using the same data, of characteristics of abusers. Findings indicate that about 24% of abusers were caregivers with disabilities, suggesting the importance of supporting caregiver health and welfare to help prevent abuse. The symposium concludes by presenting findings from a longitudinal survey of older adults aged 75+ (N=769) indicating that physical restraints use is associated with “having been diagnosed as intractable neurological diseases,” “unstable general condition or at the terminal stage,” “higher levels of care-need,” “having medical treatment,” and “family members’ long-term care burdens.”

